# Extracellular Matrix Alterations Due to Early-Life Adversity: Implications for Auditory Learning in Male Sprague–Dawley Rats

**DOI:** 10.1007/s12035-025-04690-2

**Published:** 2025-01-15

**Authors:** Aise Rumeysa Mazi, Yunus Karakoc, Cumaali Demirtas, Ugur Aykin, Mehmet Yildirim

**Affiliations:** 1 Department of Biophysics, Hamidiye Faculty of Medicine, University of Health Sciences, Selimiye Mah. Tibbiye Cad. No:38, 34668 Uskudar, Istanbul Turkey; 2 Department of Physiology, Hamidiye Faculty of Medicine, University of Health Sciences, Istanbul, Turkey

**Keywords:** Extracellular matrix, Cognitive flexibility, Childhood trauma, Early-life adversities

## Abstract

This study aimed to investigate the impact of early childhood chronic stress on the development of the brain extracellular matrix (ECM) and how alterations in the ECM following early-life adversity (ELA) affect auditory learning and cognitive flexibility. ELA was induced through a combination of maternal separation and neonatal isolation in male Sprague–Dawley rats, and the success of the ELA model was assessed behaviorally and biochemically. A cortex-dependent go/no-go task with two phases was used to determine the impact of ELA on auditory learning and cognitive flexibility. The effects of the ECM on cognition were tested via the enzymatic removal of the ECM. The molecular structure of the adult ECM was examined via immunohistochemistry. ELA impaired initial auditory learning but did not significantly affect cognitive flexibility. Hyase injection into the auditory cortex (ACx) restored initial learning. ELA rats display a reduced perineural net (PNN) and parvalbumin + cell density. Our findings reveal that ELA induces significant alterations in the ECM within the ACx, accompanied by impaired initial auditory learning. Although PNN density is already lower in ELA rats, degrading the ECM facilitates the repair of auditory learning. A reduced PNN number in ELA rats fails to enhance learning unless supplemented with Hyase injection.

**Supplementary Information:**

The online version contains supplementary material available at 10.1007/s12035-025-04690-2.

## Introduction

Adverse early-life experiences profoundly impact brain development and subsequent cognitive functions and contribute to long-lasting behavioral outcomes in adulthood [1–3]. Recent studies have indicated a potential effect of early-life adversity (ELA) on the extracellular matrix (ECM), a complex network of macromolecules surrounding neurons, in shaping neuronal plasticity and cognitive processes [4, 5].

The ECM is an important modulator of synaptic function and hence a regulator of synaptic transmission and the dynamics of neuronal connections [6–8]. The formation of mature ECM and special ECM structures called perineural nets (PNNs) commences following the end of the critical period, which is the phase of heightened synaptic plasticity during brain development [9–11]. Mature ECM is a dynamic structure, capable of remodeling in response to physical disruptions, changes in neural activity, or targeted enzymatic removal, which temporarily reduces ECM density and increases neuronal plasticity [8, 12, 13]. This process allows for increased flexibility in neuronal connections, potentially improving learning and memory capacity and promoting adaptive reorganization in the brain.

Disruption of the ECM structure and organization has been associated with several neuropsychiatric disorders, including those related to ELA [14]. ELA alters brain regions, such as the prefrontal cortex, hippocampus, and amygdala, at the molecular and cellular levels. These alterations, in turn, manifest as cognitive impairments, attention deficits, memory deficits, and cognitive inflexibility [15, 16].

While the precise mechanisms underlying the impact of early-life trauma on learning, memory, and cognitive flexibility are not fully understood, recent research suggests the potential involvement of the ECM. Animal studies have revealed that the enzymatic removal of the ECM results in increased cognitive flexibility and the restoration of synaptic plasticity, similar to juvenile ECM, both in vivo and in vitro [17–19]. These results suggest that increased ECM density may impede synaptic plasticity and disrupt the balance of synaptic connectivity within brain circuits critical for cognitive flexibility.

Here, we aimed to explore the relationships among ELA, the mature ECM, and, in this context, cortical plasticity and cognitive flexibility in Sprague‒Dawley rats. Using a well-established model of early-life trauma, we assessed behavioral performance in a cognitively demanding two-way active avoidance test and assessed the role of the ECM via intracortical enzymatic removal. We also examined the ECM density in cortical regions of ELA model animals. Overall, we aimed to fill the gap in the literature regarding whether ELA affects mature ECM structure, contributing to a more comprehensive understanding of the effects of early chronic stress on brain development and the pathologies it leads to in the long term.

## Methods

### Animals

The study involved 40 male Sprague‒Dawley rats selectively bred from the original lineage obtained from Charles River Laboratories (Charles River Laboratories, Research Models and Services, Germany GmbH). The rats were housed at the University of Health Sciences, Laboratory Animals Production and Research Center, with controlled humidity (50% ± 10) and temperature (22 ± 1 °C) under a 12:12-h light‒dark cycle. The procedures were reviewed and approved by the Animal Experimentation Ethics Committee at the University of Health Sciences (protocol no.: 2021–02/02).

Several studies indicate a sex-dependent anxiety response and PNN formation following early-life adversities, such as maternal separation and neonatal isolation which are used as models for early-life adversities [4, 20, 21]. Additionally, hormonal fluctuations during the estrous cycle can influence the anxiety response and overall behavior of female rats [22–24]. To minimize the effects of these factors on behavior, only male rats were used in this study.

The sample size was determined by separate statistical power analysis for each experiment, and the largest sample size was selected as the basis for determining the number of animals used (G*Power 3.1.9.4; Cohen’s *d* = 0.5, alpha = 0.05, and power = 0.80) [25].

### Modeling Early-Life Adversity Through Maternal Separation and Neonatal Isolation

Maternal separation (MS) and neonatal isolation (NI) were used to model early-life adversities (ELA). From postnatal day (PND) 2 to 14, the rat pups from 5 litters were separated from their dams daily for 180 min, housed in a room different from their mothers, and isolated from each other by a separator [26, 27]. The temperature was adjusted to 30 ± 3 °C by a homeothermic blanket (Harvard Apparatus, Holliston, MA, USA). After PND14, the pups were returned to normal housing until they were weaned on PND23, and only males were chosen for this study. All subsequent experiments were performed during adulthood (15 weeks).

### Experimental Design

**Fig. 1:**
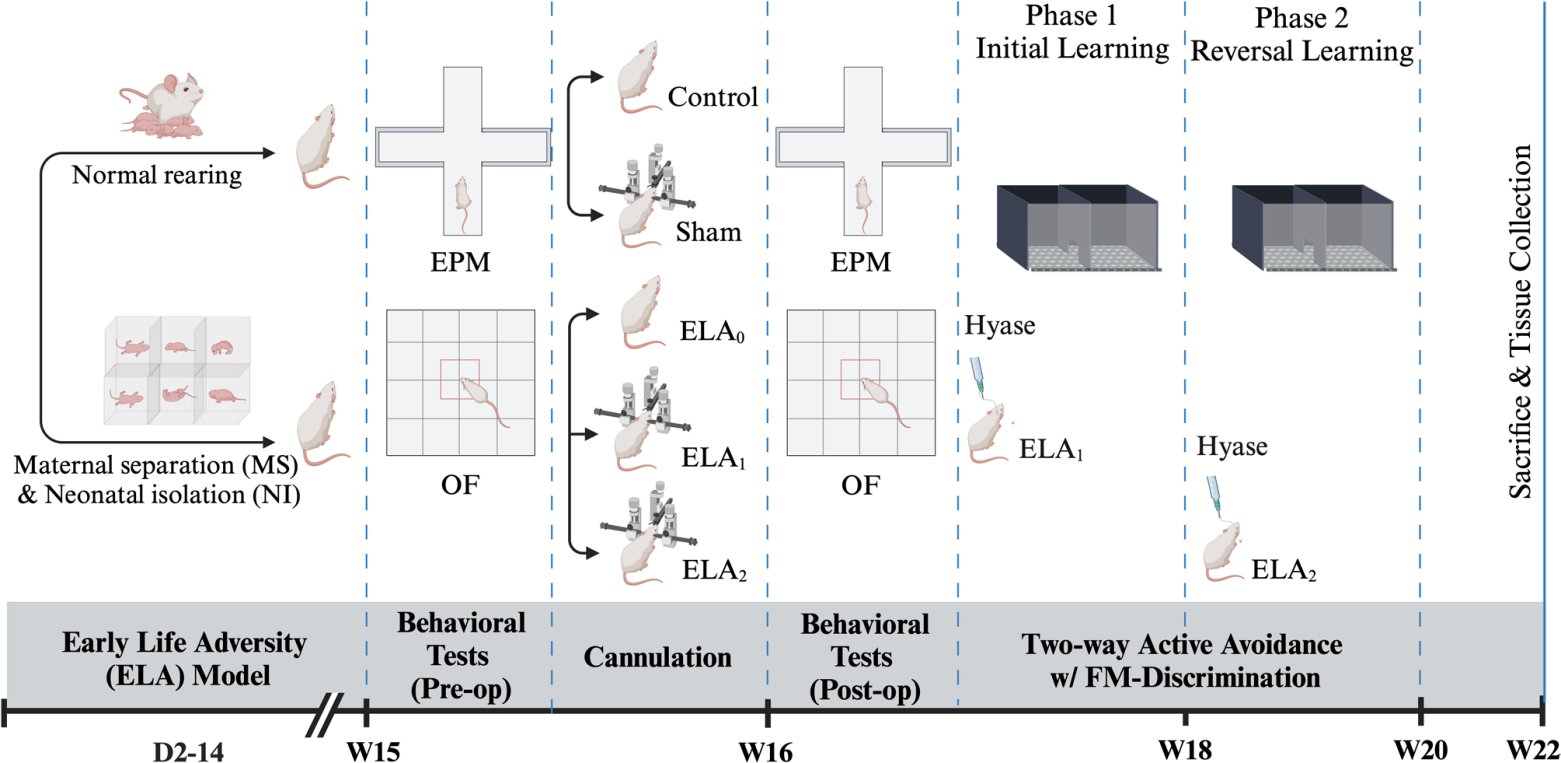
Experimental design. Workflow diagram illustrating the in vivo procedures carried out throughout the study (Created with BioRender.com)

The overall experimental design, including animal rearing, is illustrated in Fig. 1. Initial experimental groups were as follows:

- Control group (*n* = 16): normal rearing
- Early-Life Adversity group (ELA, *n* = 24): MS and NI

Immediately prior to cannulation, the control group was divided into two subgroups: the control and sham control groups. Meanwhile, the ELA group was divided into three subgroups: ELA_0_, ELA_1_, and ELA_2_. Animals were randomly assigned to groups while ensuring that the average body weight was comparable across all groups.

These new groups and treatments of animals in these groups are as follows:

- Control group (*n* = 8): normal rearing, no cannulation
- Sham control group (*n* = 8): normal rearing, cannulation
- Early-Life Adversity group (ELA_0_, *n* = 8): MS and NI and no cannulation
- Early-Life Adversity + Phase 1 Hyase group (ELA_1_, *n* = 8): MS and NI and Hyase injection before phase 1 of the two-way active avoidance test
- Early-Life Adversity + Phase 2 Hyase group (ELA_2_, *n* = 8): MS and NI and Hyase injection before phase 2 of the two-way active avoidance test

### Behavioral Tests

Elevated plus maze (EPM) and open-field (OF) tests were conducted on the same day during the diurnal phase (lights on) of the 12:12-h light–dark cycle, beginning with the OF test. Testing took place in a sound-attenuated room under consistent lighting (48 lx at maze level). Animals were acclimated to the testing room for 30–60 min prior to placement on the apparatus. Each apparatus was cleaned with a 20% ethanol solution and dried after each animal to prevent residual moisture and scent exposure. To mitigate the effects of same-day testing and test order, we maintained a 3-h interval between tests, allowing for recovery and reducing variability. Testing environments were standardized in lighting, noise, and other contextual factors to minimize external influences on behavior.

#### Elevated Plus Maze Test

The elevated plus maze was used to examine the anxiety behavior of ELA (total *n* = 24) and control (total *n* = 16) rats [2, 28]. The number of entries into the open arms and the distance traveled in the open arms were detected by ANY-Maze object tracking software for 300 s (ANY-Maze Video Tracking System, Stoelting Europe, Dublin, Ireland). The anxiety index was calculated via the following formula: 1 − [([Time in open arms/Test duration] + [number of entries into open arms/Total number of entries into arms])/2] [29]. The test was repeated after cannulation to examine the effect of the procedure on the stress response.

#### Open Field Test

The open-field test was used to compare the locomotor activity of the ELA (total *n* = 24) rats with that of the control (total *n* = 16) rats. The animals were placed into the open-field test setup with dimensions of 100 × 100 × 30 cm split into 16 equal squares from a predetermined corner, and their behaviors were recorded for 300 s (ANY-Maze Video Tracking System, Stoelting Europe, Dublin, Ireland). The total distance traveled, number of entries into the center, and time spent in the center were considered indicators of locomotor activity and anxiety [30–32]. The test was repeated after cannulation to examine the effects of the procedure on locomotor activity.

### Intracortical Cannulation and Injection

#### Cannulation

The rats in the ELA_1_, ELA_2_, and sham control groups were implanted with i.c.v. guide cannulas (C313GRL/SPCguide 22GA, Plastic One Inc., Roanoke, VA, USA) bilaterally under 90 mg/kg (i.p.) ketamine hydrochloride (Ketalar, Pfizer, Istanbul, Turkey) and 10 mg/kg (i.p.) xylazine hydrochloride (Xylazinbio, Biyoveta, Istanbul, Turkey) in combination, via a stereotaxic apparatus (Stoelting Instruments, Wood Dale, IL, USA). Cannulas were placed in the temporal cortex (AP + 2.0 mm, ML ± 3.5 mm, and DV + 3.5 mm by bregma). Coordinates were determined according to the rat brain atlas (Paxinos & Watson, 1986) and validated by implantation into same-age rats that were not included in the main study. The guide cannula was closed with a stainless-steel wire dummy cannula (C313DC/SPCdummy 0.014/0.36 mm, Plastic One Inc., Roanoke, VA, USA) when not injected. The infusion cannulas were attached to the skull via three stainless-steel screws and dental acrylic. Although cannulation can cause short-termr ECM disruption, this effect does not persist long term [33, 34]. Following cannulation, the rats were housed in plexiglass cages for a recovery period of 1 week, allowing the brain ECM to return to baseline levels. Additionally, a sham control group was included to account for any temporary ECM changes due to cannulation.

#### Hyase Injection

ELA_1_ rats were injected (i.c.) with the ECM-degrading enzyme hyaluronidase (Hyase, Sigma-Aldrich HX0514) before the initial discrimination phase, and ELA_2_ group rats were injected before the reversal learning phase of the two-way active avoidance test. Microinjections were administered bilaterally with a 25 µl Hamilton microinjector (702 N; Hamilton Bonaduz, Switzerland) through previously implanted cannulas and a flexible polyethylene tube into the auditory cortex (ACx) (AP + 2.0 mm, ML ± 3.5 mm, and DV + 3.5 mm by bregma), delivering a volume of 10 ml (750 U) of Hyase solution. The use of Hyase in this study was preferred over other enzymes due to its distinct advantage of allowing the ECM to return to its normal state within approximately 2 weeks after administration into the brain [35, 36]. This characteristic reduces the risk of the enzyme’s effects extending into subsequent experimental phases, which was critical for our experimental design.

### Two-Way Active Avoidance Test with FM Discrimination

**Fig. 2:**
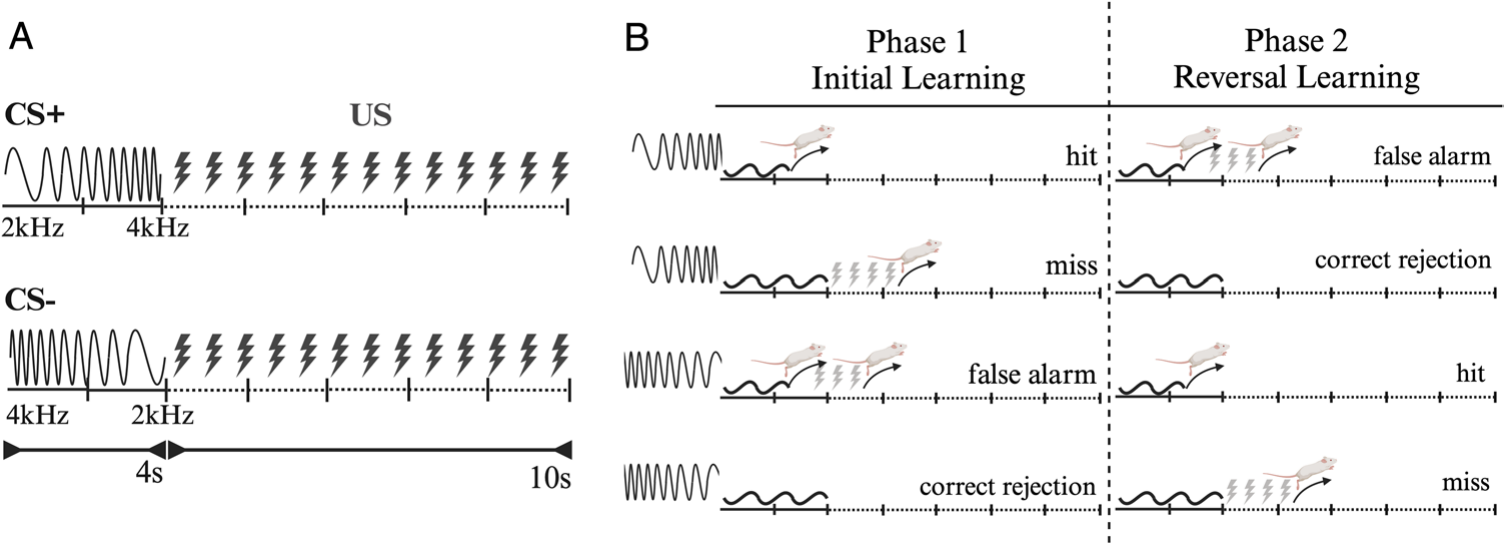
Task design (**A**) and behavioral outcomes (**B**) for the CS + and CS − trials in a two-way active avoidance test with a frequency-modulated discrimination task (Created with BioRender.com)

Adult Sprague–Dawley rats were trained once a day in a two-compartment shuttle box (Ugo Basile, Comerio VA, Italy) to discern the direction of linear frequency modulation employed as conditioned go/no-go stimuli (70 dB; CS + : 2–4 kHz; CS–: 4–2 kHz; duration: 4 s). Individual foot shock intensities (0. 1–0.4 mA) were adjusted and delivered through a metal floor grid as the unconditioned stimulus (US) (Fig. 2A).

The training was conducted in daily sessions of 40 trials (20 CS + , 20 CS −) presented in randomized order after fixed intertrial intervals of 15 s. A compartment change after CS + onset within 4 s was classified as a “hit” response. The absence of a hit response was counted as a “miss,” and the US of 10 s was immediately delivered following the CS + and terminated by a change in the compartment (Fig. 2B).

For the CS- trials, a compartment change within 4 s was classified as a “false alarm” response, and the US was applied for up to 10 s following the movement. The US was not delivered after the CS − if the animal stayed in the compartment, and the response was considered “correct rejection.” Hit rates (CR +) and false alarm rates (CR −) were calculated as the percentage of hits and false alarms in each 40-trial session. After 14 days of the initial learning phase, the rats were trained with a reversed contingency as the reversal learning phase.

### Sacrifice and Tissue Collection

The rats were sacrificed by cervical dislocation under 90 mg/kg (i.p.) ketamine hydrochloride (Ketalar, Pfizer, Istanbul, Turkey) and 10 mg/kg (i.p.) xylazine hydrochloride (Xylazinbio, Biyoveta, Istanbul, Turkey) in combination 2 weeks after the experiments.

Blood samples were collected through an insulin injector from cardiac blood and allowed to coagulate in a microcentrifuge for 30–60 min. The supernatants were collected after centrifugation at 1500 × g for 10 min.

The brains were rapidly removed, fixed in 4% PFA for 24 h, and washed 3 × 10 min in PBS before being cryoprotected in 15% and 30% sucrose. After the tissues sank in sucrose, they were frozen and embedded in a matrix (Bioblock).

### Serum Corticosterone Measurement

The serum corticosterone (CORT) concentration was determined via competitive ELISA (CEA540Ge, USCN Life Science Inc., Wuhan, China). Tests were performed according to the manufacturer’s instructions.

### Immunohistochemistry

The brain tissues were cut into 50-µm-thick coronal sections via a cryostat (Cryostar NX50, Thermo Scientific) and collected in PBS-containing wells. Four to six sections containing the temporal cortex were stained according to the free-floating tissue staining protocol of Potts et al. with necessary adjustments [37]. The tissue sections were washed and blocked in PBS supplemented with 0.3% Triton-X (#1,003,243,275, Sigma–Aldrich), 5% goat serum (Cat# GOA-1B, Capricorn Scientific) for 1 h at RT before being stained for 2 h at 37 °C in a primary antibody cocktail containing anti-PV (1:200, Sigma‒Aldrich Cat# SAB4200545, RRID: AB_2857970) and WFA-FITC (1:200, Invitrogen Thermo Fisher Scientific, Cat# L32481, RRID: AB_308666666). The sections were washed 3 × 10 min in PBS, transferred to secondary antibody (goat anti-mouse Alexa Fluor® 594, 1:500, Abcam, Cat#ab150120, RRID: AB_2631447) and incubated for another 2 h at 37 °C. The tissue sections were mounted onto slides and covered with mounting medium (Fluoroshield, Cat# F6182; Sigma Aldrich).

All imaging and analyses were performed by an investigator blind to the experimental groups. Images were captured with an Axiocam-105 camera connected to a Zeiss Axio Vert.A1 fluorescence microscope and Zen Blue 2 software (RRID: SCR_013672). For analysis, three images were taken from the ACx and motor cortex (MCx) of each animal. Image analysis was conducted via ImageJ (https://imagej.net/ij/, RRID: SCR_003070) and CellProfiler Image Analysis Software (www.cellprofiler.org, RRID: SCR_007358).

### Statistical Analysis

Data are presented as medians with interquartile ranges (IQRs) and means ± SEMs depending on the normality of the distribution. SPSS (RRID:SCR_002865) and Jamovi (RRID:SCR_016142) software were used for the analyses. Statistical significance levels are expressed as *p < 0.05, **p < 0.01, and ***p < 0.001.

Serum CORT levels and the results of the EPM and OF tests were compared between the control group (*n* = 16) and the ELA group (*n* = 24). Owing to the nonnormal distribution according to the Shapiro–Wilk test, comparisons between these two groups were performed via the Mann‒Whitney *U* test. Histological analyses were also performed between these two groups via paired-sample Student’s *t* tests.

FM discrimination was evaluated in five groups: the control (*n* = 8), sham control (*n* = 8), ELA_0_ (*n* = 8), ELA_1_ (*n* = 8), and ELA_2_ (*n* = 8) groups. Daily learning performance was calculated as the difference between hit rates and false alarm rates, where hit rates = hits/number of trials and false alarm rates = false alarms/number of trials. Discrimination sensitivity was assessed via d′ values via signal detection theory [38], where a d′ of 1.0 indicates a signal discrimination strength of one standard deviation above the noise level. Discrimination performance within each group was evaluated via the Wilcoxon signed-rank test. Learning and discrimination performance differences between groups were analyzed via mixed-model ANOVA (with Greenhouse–Geisser and Huynh–Feldt correction of sphericity when necessary) since ANOVA is generally robust to nonnormal data when sphericity is met [39, 40].

## Results

### Biochemical and Behavioral Characterization of ELA

**Fig. 3:**
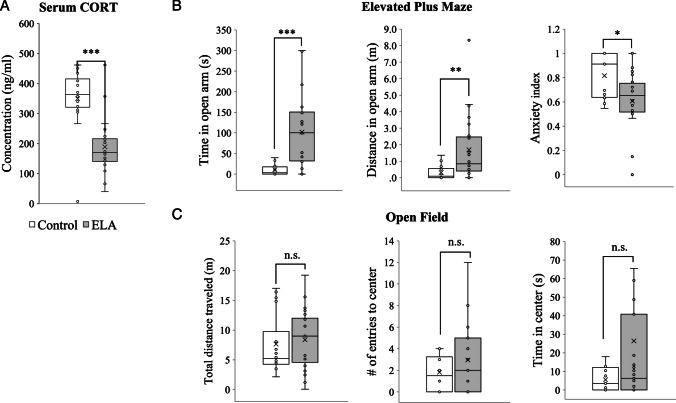
Stress response following ELA in adulthood. **A** Blood corticosterone levels in ELA rats compared with those in control rats. **B** Activity in the open arms and anxiety indices during the elevated plus maze test. **C** Mobility and activity in the center zone during the open field test (Mann‒Whitney *U* test, *p < 0.05, **p < 0.01, and ***p < 0.001)

To assess the physiological repercussions of ELA, we investigated serum corticosterone (CORT) levels via ELISA. The results revealed a significant decrease in the serum CORT level in the ELA group compared with the control group (*p* < 0.001, Fig. 3A).

Elevated plus maze (EPM) and open-field (OF) tests were conducted to assess the animals’ anxiety responses and locomotor activity. EPM and OF tests were first conducted before cannulation to establish baseline behavior, and these results are presented in Fig. 3B. The tests were then repeated after cannulation to evaluate the impact of the operation on behavior in the ELA_1_ and ELA_2_ injection groups as well as the sham control group, with these results shown in the supplementary material (Fig. S1).

In the EPM test, the time spent (*p* < 0.001) and distance traveled in the open arms (*p* < 0.01) were significantly greater, whereas the anxiety index was significantly lower (*p* < 0.05) in the ELA group than in the control group (Fig. 3B).

The open-field test revealed no significant difference in locomotor activity between the groups. Similarly, there was no significant change in the number of entries or the time spent in the center area (Fig. 3C).

EPM and OF tests were repeated after cannulation to evaluate the impact of the operation on behavior in the ELA_1_ and ELA_2_ injection groups and the control group. Following cannulation, the ELA_0_ and ELA_1_ animals spent less time in the open arms (*p* < 0.05) during the elevated plus maze test (Fig. S1A). The ELA_2_ group presented a decrease in the distance traveled in the open arms (*p* < 0.05), whereas the anxiety index did not change significantly. In the open-field test after cannulation, the ELA_0_ and ELA_2_ groups presented decreased center entries (p_ELA0_ = 0.033, p_ELA2_ = 0.049) and time spent in the center (p_ELA0_ = 0.028, p_ELA2_ = 0.093). The cannulation groups (sham control, ELA_1_, and ELA_2_) presented an overall decrease in the total distance traveled (Fig. S1B). The immobility time decreased significantly in all the groups, but the difference was more pronounced in the ELA groups.

### Initial Learning and Reversal Learning During the FM Discrimination Task

**Fig. 4:**
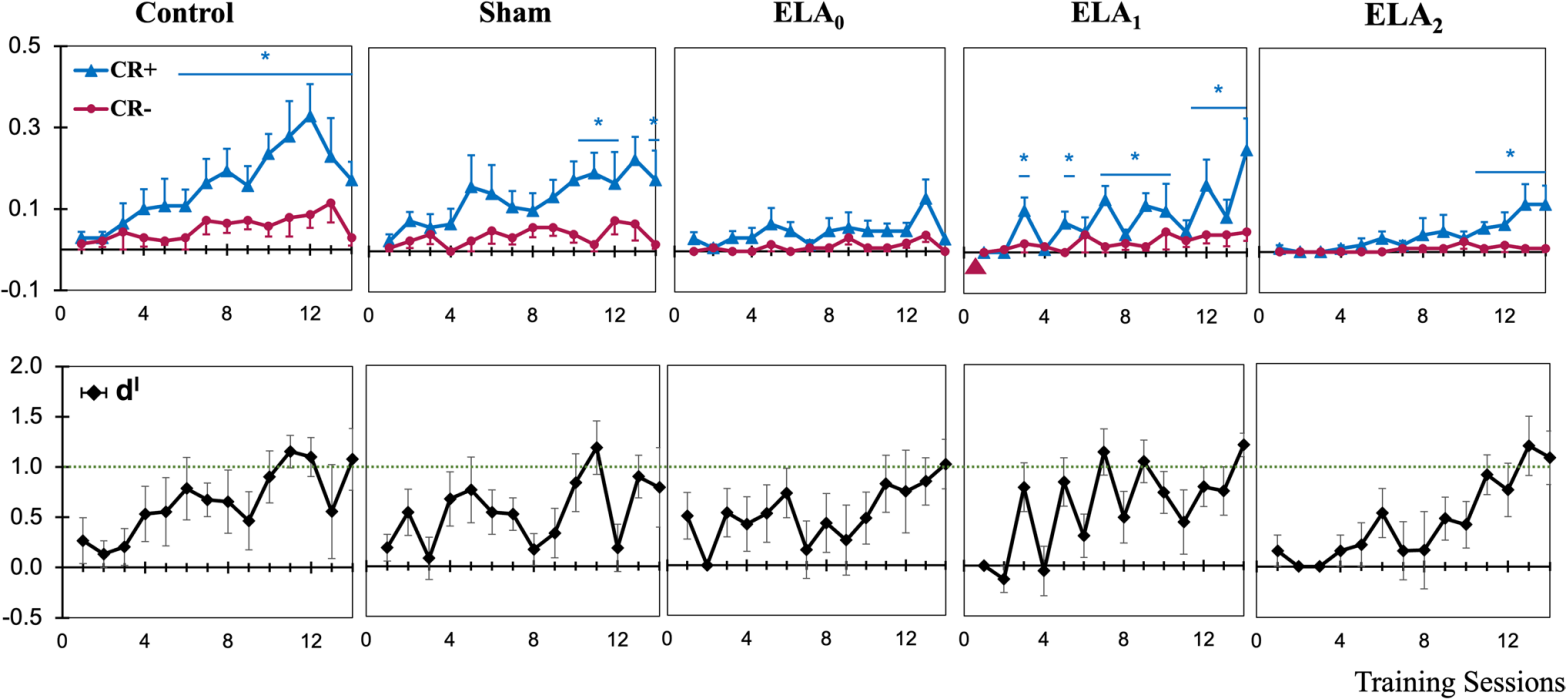
Comparison of learning curves for Phase 1-Initial learning phase. CR + and CR- learning curves (upper) and d′ learning curves (lower) of the FM discrimination task plotted as a function of training sessions (once per day) for all groups (Wilcoxon signed-rank test, *p < 0.05)

The first phase of the two-way active avoidance test was designed to investigate whether ELA affects initial acquisition learning. Only ELA_1_ rats received a Hyase injection before the experiment. During the initial acquisition phase, there was a significant increase in the hit rates from day 7 for the control rats (*W* = 0, *p* < 0.05). The ELA rats, except those in the Hyase-injected group ELA_1_, did not reach a significant hit rate or discrimination level until the last days of phase 1 (Fig. 4). A mixed-model ANOVA revealed a significant interaction effect between the groups and sessions on performance, *F*(52,338) = 1.44, *p* = 0.031. Further analysis using Tukey’s post hoc test indicated that the ELA_0_ group (*p* = 0.040) and ELA_2_ group (*p* = 0.011) displayed significant differences in performance compared with the control group (Fig. 6A, C). ELA_1_ rats completed the first phase, with discrimination performance similar to that of the control group (*p* = 0.126) (Fig. 6B).

**Fig. 5:**
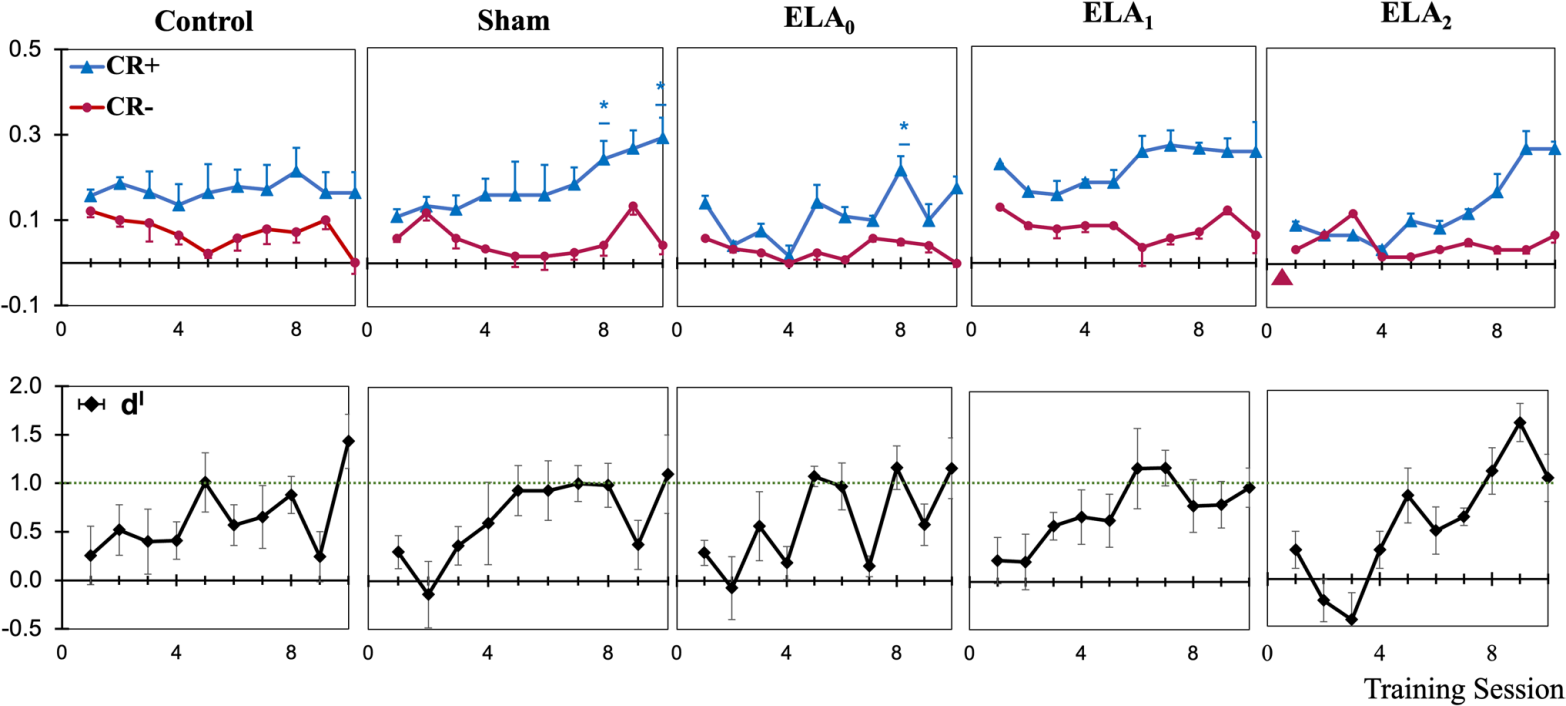
Comparison of learning curves for the phase 2-reversal learning phase. CR + and CR- learning curves (upper) and d′ learning curves (lower) of the FM reversal discrimination task plotted as a function of training sessions (once per day) for all groups (Wilcoxon signed-rank test, *p < 0.05)

**Fig. 6:**
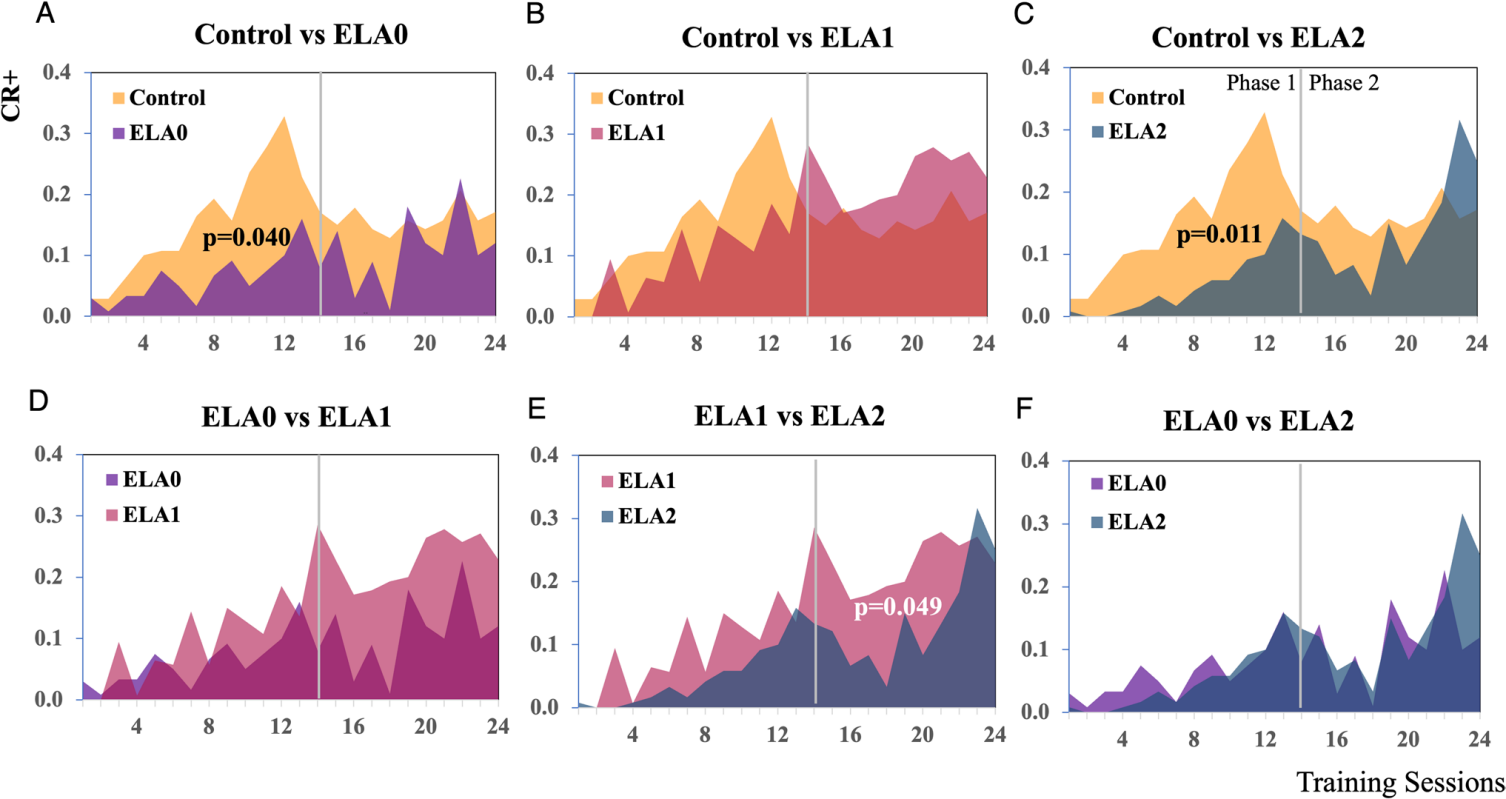
Comparison of learning curves between groups. CR + and CR − differences in the FM discrimination task plotted as a function of training session (once per day). The gray lines indicate the changes from phase 1 to phase 2

The second phase of the experiment was intended to determine the cognitive flexibility of the ELA rats. ELA_2_ was injected with Hyase in the ACx before the phase started. Evaluation of the second phase revealed that none of the groups achieved statistically significant discrimination performance over three consecutive days (Fig. 5). The interaction effect between groups and sessions on performance was not significant (*F*(36,234) = 0.852, *p* = 0.659). Further analysis of the second phase using Tukey’s post hoc test revealed no significant difference in the learning and discrimination performance between the control and ELA groups (Fig. 6A–C). Compared with the control and ELA_0_ groups, the Hyase-injected ELA_2_ group did not significantly differ in performance (Fig. 6C, E). However, the ELA_1_ group performed significantly better than the ELA_2_ group (0 = 0.049) (Fig. 6D).

### Immunofluorescence Staining of the ECM

**Fig. 7:**
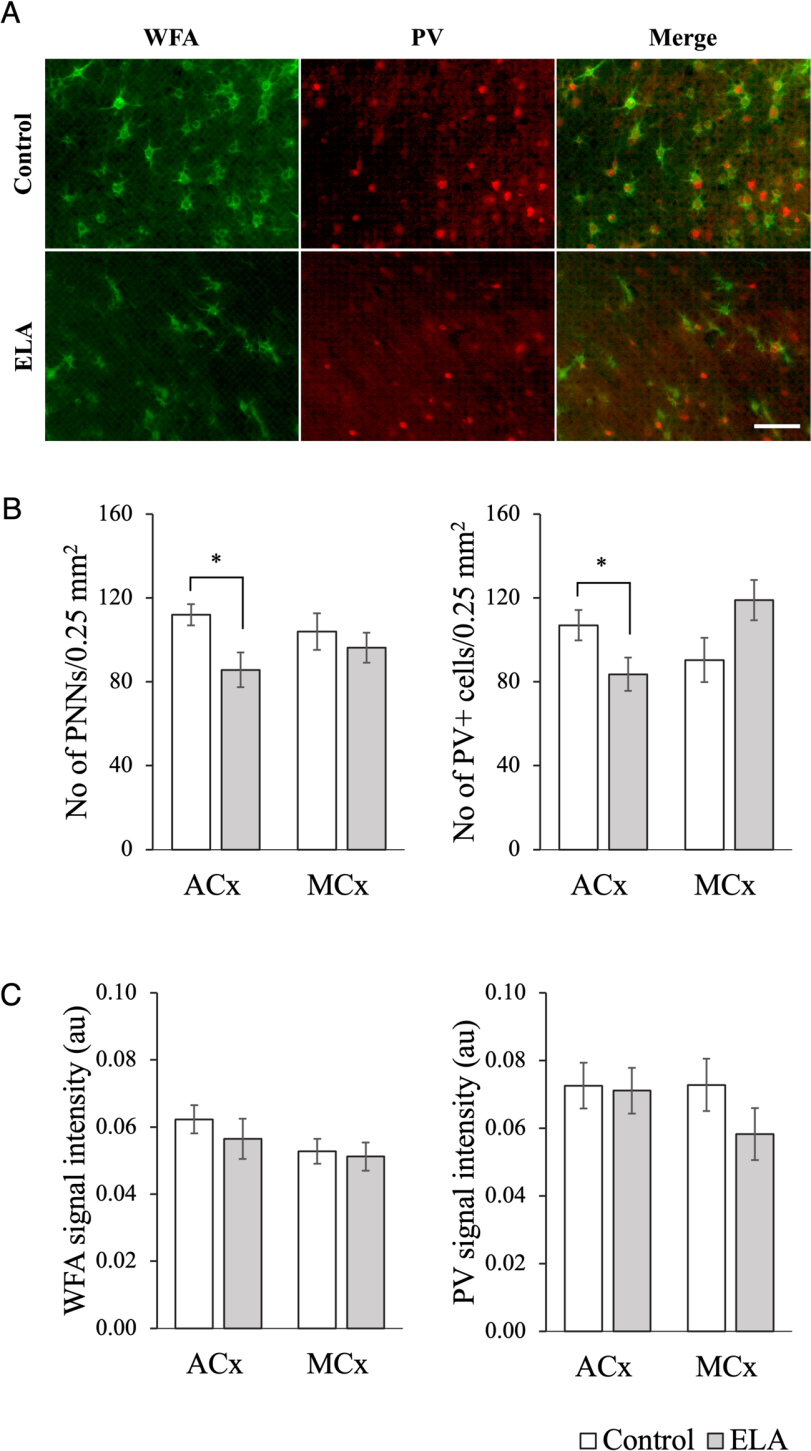
ECM density and signal intensity monitored by immunohistochemical analysis. **A** Example of WFA and PV fluorescein staining of the ACx. **B** Quantitative analysis of the normalized WFA + PNN and PV + cell density. **C** Quantitative analysis of the normalized WFA and PV fluorescence signal intensities (Student’s *t* test, *p < 0.05, scale bar = 100 µm)

We used immunofluorescence staining to investigate the number of PV + cells and WFA + PNNs within the ACx and motor cortex (MCx) of the ELA rats (Fig. 7A).

In the ACx, there was a statistically significant decrease in the number of PNNs in the ELA group (*p* = 0.012, Fig. 7B), although the mean WFA intensity remained the same (Fig. 7C). Similar results were found in the MCx, although the decrease in the number of PNNs was not significant (*p* = 0.366).

The number of PV + cells in the ELA rats decreased significantly in the ACx (*p* = 0.040) and increased significantly in the MCx (*p* = 0.059). PV signal intensity displayed no change in the ACx and a decrease in the MCx, albeit the difference was statistically insignificant (*p* = 0.147).

## Discussion

In the present study, we explored the intricate relationships among early-life adversities, ECM dynamics, and auditory learning, providing insights into our understanding of the lasting effects of childhood experiences on brain function and offering potential avenues for therapeutic intervention, such as ECM degradation. ELA rats did not display elevated anxiety, as indicated by serum CORT levels and behavioral tests, unless they were presented with an additional stressor. In the initial learning phase of the FM discrimination task, the ELA rats exhibited impaired learning. In contrast, control rats and ELA rats injected with hyaluronidase (Hyase) presented significant learning, suggesting a negative impact of ELA on initial learning, which was subsequently improved by ECM removal. However, during the reversal learning phase, no clear difference was observed between the groups, suggesting preserved cognitive flexibility. Immunofluorescence staining revealed significant alterations in ECM molecular structures, revealing a pronounced decrease in the number of WFA + PNNs in the auditory cortex (ACx) of ELA rats, accompanied by distinct changes in parvalbumin (PV) + cell numbers, with no significant alterations in the signal intensity of the eighter WFA or PV. Despite the previous reduction in the ECM caused by ELA, Hyase injection before the initial discrimination phase restored learning performance in ELA rats, highlighting the role of the ECM in modulating cognitive functions and suggesting complex regional dynamics in response to early-life stress. Injection before the reversal learning phase did not result in significant differences, indicating varying effects of ECM removal across different cognitive processes.

Most studies that utilized the combination of maternal separation and neonatal isolation as a model of ELA reported an increase in basal and stress-induced CORT levels as well as increased anxiety in behavioral tests such as the EPM and OF tests [41–43]. Nevertheless, the relationship between ELA and the adulthood stress response is complex and can vary on the basis of factors such as the timing, duration, and nature of childhood adversity. Several studies have also suggested that ELA can increase resilience to stress in adulthood [44, 45]. In addition, some meta-analyses reported that unconditional behavioral tests, such as the EPM test, are inadequate for detecting stress responses caused by ELA, especially in the absence of secondary adulthood stressors [46]. This finding is also evident in our results, which revealed that indicators of anxiety increased in the ELA group in the second round of the behavioral tests after the second stress factor, which are the initial behavioral tests and cannulation. An increase in the anxiety response was also observed in ELA_0_ animals, which had not undergone surgical intervention, indicating that the increased anxiety response is attributed to repeated stressful conditions rather than surgical intervention. It is important to note that repeated testing, however far apart like in our study design, has consequences such as increased open-arm avoidance and decreased locomotor activity [47, 48]. However, the control and sham groups showed no significant differences between pre-op and post-op tests, suggesting that the heightened anxiety response in ELA groups is not simply due to repeated testing or reduced novelty. This aligns with findings that ELA can increase sensitivity to later stressors, indicating that the observed anxiety increase in ELA groups may be due to this sensitization rather than repeated testing alone.

A history of ELA negatively impacts the development of brain regions crucial for learning and memory, including the prefrontal cortex, orbitofrontal cortex, and hippocampus [1, 5, 16, 49]. Additionally, ELA induces amygdala hyperactivation, potentially disrupting cognitive processes and synaptic plasticity in cortical regions, causing impaired learning and memory [50–52]. Frequency-modulated (FM) discrimination tasks, especially in two-way paradigms, require a high level of ACx plasticity. In line with our hypothesis, the ELA group exhibited poorer learning and discrimination performance in the initial acquisition phase than the control and sham control groups did, unless they received an intracortical Hyase injection beforehand. Our results also corroborate previous findings where the endogenous or exogenous degradation of the ECM improved synaptic plasticity and learning [11, 53–55].

During the second phase of the experiment, which measures cognitive flexibility through reversal learning, the experimental groups exhibited similar discrimination performances on the basis of the reversed contingency and required an extended training period. Reversal learning is a more complex mechanism than initial learning. It consists of two stages: discarding the strategy acquired during the initial learning phase and developing a new discrimination strategy on the basis of the new contingency [56]. A closer look into the data revealed that the control, sham control, and ELA_1_ groups, which had faster learning trajectories during phase 1, displayed matching learning patterns with higher false alarm rates during the reversal learning phase. On the other hand, ELA_0_ and ELA_2_ presented a learning performance resembling the initial learning phase of the remaining groups. Taken together, these results suggest that the animals that reached a successful discrimination level in phase 1 struggled to discard the initial behavioral strategy during phase 2, whereas the rest learned the reverse contingency as it was the initial condition. Although the statistical analysis does not indicate an effect of ELA or ECM degradation on cognitive flexibility, the stable increase in hit rates suggests that an extended training period could lead to a different result.

PNNs are commonly recognized for their ability to restrict synaptic plasticity, which led to the hypothesis that ELA animals would have a denser PNN structure [57, 58]. Since ECM development also regulates the maturation of PV interneurons, a similar alteration in PV + cell density was anticipated [59]. Contrary to our expectations, the IF data revealed that adult rats with a history of ELA had fewer WFA + PNNs and PV + cells in the ACx. In contrast, the WFA or PV signal intensity did not differ significantly from that of the control. Although the PNN density and PV + cell number are affected by adversity, the direction of change varies depending on the type and timing of the adverse experience and the particular brain region involved [14, 60]. PV + interneurons are crucial in controlling the timing and synchrony of neuronal activity, improving the precision of auditory processing, and enabling the discrimination of auditory patterns [61]. Although several studies have reported that ELA does not significantly alter PV + neuron density [4, 51], our data, which revealed a decrease in PV + cell density accompanied by learning deficiency in ELA animals, align with the role of PV + neurons. While a reduction in the mature ECM enhances synaptic plasticity, it also increases the vulnerability of neurons to damage and threatens the stability of synaptic connections. An elaborate study by Gildawie et al. revealed that PNN density is reduced in the prelimbic (PrL) and infralimbic (IL) prefrontal cortices after maternal deprivation throughout juvenility, whereas it is increased in the amygdala until adulthood, when it returns to normal levels [4]. Another study revealed decreased WFA intensity in the CA1 region of the hippocampus, dorsal anterior cingulate cortex (ACC), prelimbic cortex, and MCx in mice with a history of juvenile stress [62].

Given the evidence suggesting that PNNs restrict plasticity and cognitive flexibility, our results showing that the reduced WFA density of ELA animals coincides with impaired cortical learning may seem paradoxical. However, it is important to note that WFA is not a pan-PNN marker and mainly visualizes sulfation patterns. Labeling other mature ECM molecules may provide a more comprehensive understanding of the relationship between ELA and the ECM. In addition, synaptic plasticity and learning are not mechanisms that can be explained by a single factor, as numerous studies over the years have confirmed.

Hyase degrades ECM by cleaving hyaluronic acid (HA) backbone; a change can be readily visualized using anti-WFA staining. Previous studies have shown the molecular and behavioral consequences of ECM degradation by Hyase in rodents including Sprague–Dawley rats [18, 36, 63, 64]. In this study, although we administered Hyase injections into the ACx to assess the impact of ECM modulation on auditory learning and reversal learning, we did not conduct direct visualization of the ECM immediately following Hyase injection. Instead, we focused on assessing the impact of Hyase on auditory learning. The fact that Hyase injection facilitated auditory learning in ELA animals suggests that ECM degradation may be utilized as a therapeutic tool to improve cognitive function after childhood trauma. ECM molecules are continuously broken down and rebuilt within the homeostatic balance of the brain. While how the combination and density of these molecules impact brain function is still under investigation, it is evident that there is a finely calculated balance regarding the ECM. For example, excessive ECM degradation can lead to the brain entering an epileptic state [64–66]. On the other hand, minimal degradation can enhance synaptic plasticity [58, 67, 68] or impair fear-based memory [50]. Correctly determining in which direction and to what extent this shift is beneficial is essential when the ECM is modified through enzymatic degradation or genetic interventions.

It is important to acknowledge that this study utilized only male rats to investigate the effects of ELA on auditory learning and the dynamics of ECM, which was an intentional decision aimed at reducing potential variability in behavioral outcomes. The active avoidance protocol employed in our study required repeated testing over 25 days, which would overlap with different phases of the estrous cycle in female rats, potentially impacting their performance due to hormonal fluctuations. Incorporating female animals in subsequent research could provide valuable insights into the role of sex differences in the impact of ELA on cognitive performance and ECM behavior.

In conclusion, we propose that ELA alters both mature ECM and auditory learning but not necessarily through the same mechanism. Additional labeling of other ECM molecules is needed to provide a better understanding. Nevertheless, the ECM-degrading enzyme Hyase seems to be a promising therapeutic agent. The specific mechanisms through which the ECM influences cognitive functions warrant further exploration. Owing to technical limitations, which result in a shorter training period for reversal learning, determining the impact of ELA on cognitive flexibility is challenging. However, while still speculating, it is possible that ELA could result in reduced cognitive flexibility.

## Supplementary Information

Below is the link to the electronic supplementary material.

ESM 1 (PDF 212 KB)

## Data Availability

No datasets were generated or analysed during the current study.
