# Supplementary material for: Extracellular Matrix Alterations Due to Early-Life Adversity: Implications for Auditory Learning in Male Sprague–Dawley Rats
Source: Mol Neurobiol. 2025 Jan 15;62(5):6490–502. doi: 10.1007/s12035-025-04690-2 (PMC11953085; doi:10.1007/s12035-025-04690-2)
Supplement: Supplementary file 1 — (PDF 212 KB) [file 12035_2025_4690_MOESM1_ESM.pdf]

Article Title: Extracellular Matrix Alterations Due to Early Life Adversity: Implications for Auditory Learning in Male Sprague-Dawley Rats

Journal Name: Molecular Neurobiology

Authors: Aise Rumeysa Mazi, Yunus Karakoc, Cumaali Demirtas, Ugur Aykin, Mehmet Yildirim

Correspondence: Aise Rumeysa Mazi

Department of Biophysics, Hamidiye Faculty of Medicine, University of Health Sciences, Istanbul, Turkiye

E-mail: aiserumeysa.mazi@sbu.edu.tr

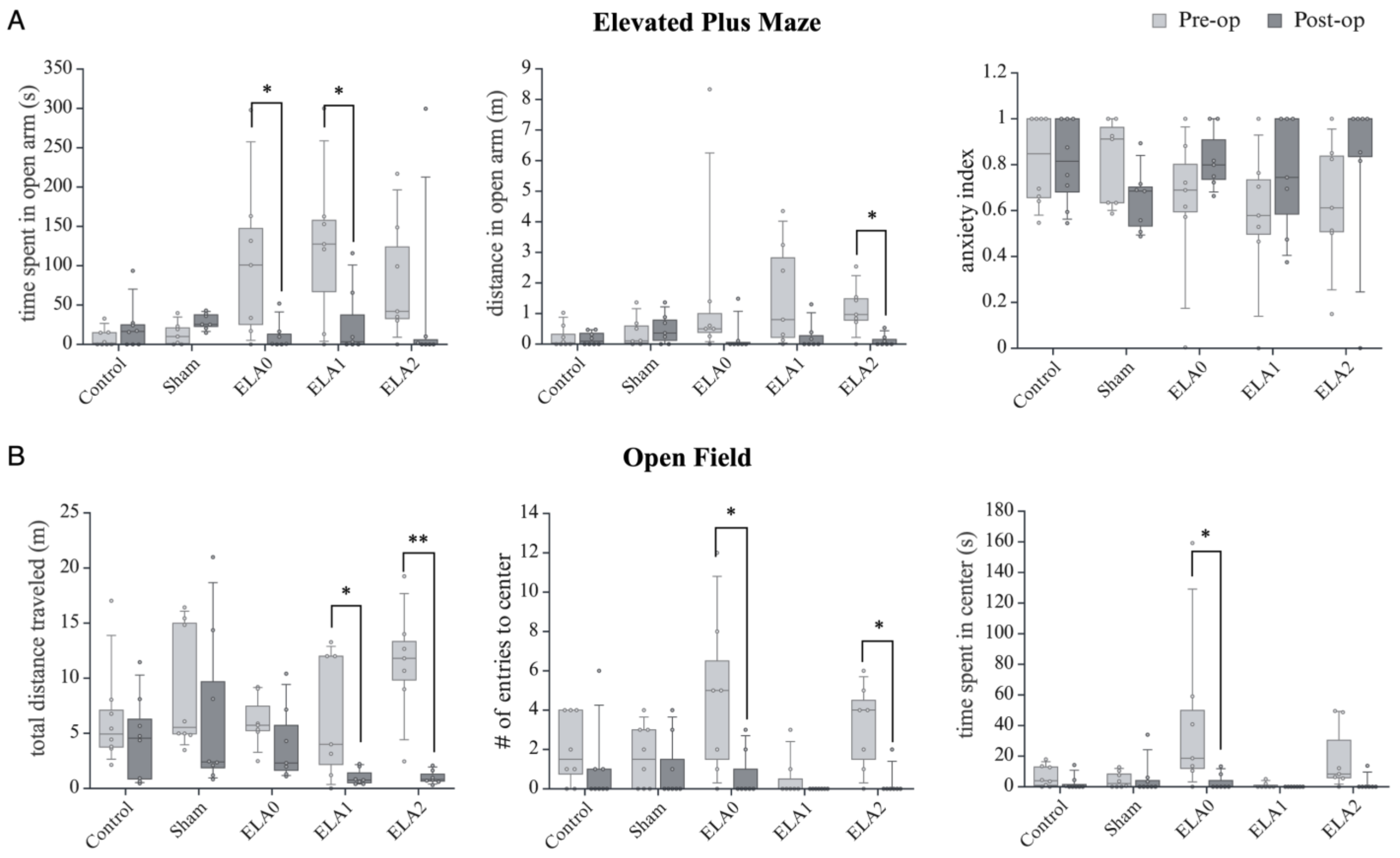

Supp Fig. 1: Effects of intracortical cannulation on anxiety and mobility in the elevated plus-maze and open field tests. (A) Activity in the open arms and anxiety indices during the elevated plus maze test before and after cannulation. (B) Mobility and activity in the center zone during the open field test before and after cannulation (Wilcoxon W, *p<0.05, and **p<0.01)
